# Construction and validation of a preterm birth risk assessment model using fuzzy analytic hierarchy process

**DOI:** 10.17305/bjbms.2021.6431

**Published:** 2021-10-04

**Authors:** Stavroula Barbounaki, Antigoni Sarantaki

**Affiliations:** Department of Midwifery, Faculty of Health and Caring Sciences, University of West Attica, Athens, Greece

**Keywords:** Preterm birth, fuzzy multi-criteria analysis, fuzzification, risk assessment, decision making

## Abstract

Preterm births account for almost 1 million deaths globally. The objective of this study is to develop and evaluate a model that assists clinicians in assessing the risk of preterm birth, using fuzzy multicriteria analysis. The model allows experts to incorporate their intuition and judgment into the decision-making process and takes into consideration six (6) risk dimensions reflecting the socio-economic, behavioral and medical profile of pregnant women, thus adopting a holistic approach to risk assessment. Each risk dimension is further analyzed and measured in terms of risk factors associated with it. Data were collected from a selected group of 35 experts, each one with more than 20 years of obstetric experience. The model criteria were selected after a thorough literature analysis, so as to ensure a holistic approach to risk assessment. The criteria were reviewed by the experts and the model structure was finalized. The fuzzy analytic hierarchy method was applied to calculate the relative importance of each criterion and subsequent use of the model in assessing and ranking pregnant women by their preterm risk. The proposed model utilizes fuzzy logic and multicriteria analysis. It addresses the multifactorial nature of decision making when assessing the preterm birth risk. It also incorporates the obstetricians’ intuitive judgment during risk assessment, and it can be used to classify cases based on their risk level. In addition, it can be applied to evaluate the risk of individual cases in a personalized manner. The proposed model is compared and validated for its predictive value against judgments made by experts.

## INTRODUCTION

Preterm birth has long been recognized as a primary cause of death, in children younger than 5 years of age [1,2]. According to the WHO, preterm births are deliveries that occurred earlier than 37 weeks of gestation [3]. The UN aims to eradicate all preventable causes of death, in children younger than 5 years, by 2030, since they account for almost 1 million deaths globally [4]. More than 84% of the total preterm births worldwide (that accounts for about 15 million deaths), happen between 32 and 36 weeks of gestation [5]. Thus, making accurate and timely assessments of the preterm birth risk (PBR), allows obstetricians and midwives to take all necessary measures to prevent a preterm labor and to avoid all repercussions associated with it.

The complexity of the PBR assessment is reflected by its multifactorial nature [6-20]. Many studies have identified several factors affecting the PBR, such as gestational age [1,21], history of preterm birth [22,23], short cervix [24], infection [1,24], short inter pregnancy interval [1], low maternal education [23], low body mass index [20,23], and ethnic origin [5]. Risk assessment is a decision-making process that can be investigated from a rational approach or an intuitive perspective [25]. The role of intuition has also been recognized, with many researchers praising the high quality and accuracy of intuitive judgments and its privilege to rational decision making [26-28].

Socio-economic factors may also influence PBR. There is only a 50% chance of survival for a baby born at 32 weeks, due to lack of available resources and poor quality of expert support in low-income countries, as opposed to the economically advanced, where babies born as early as 24 weeks, have survival chances that reach 50% [5]. In addition, behavioral factors such as smoking, alcoholism, substance use [1,20,22,23], as well as gynecologic (medical) history [23,24], induced abortion [1,20,23], demographics, periodontal disease [22,23], pregnancy complications, maternal vitamin D deficiency, vaginal bleeding, polyhydramnios [23], depression, stress [20,23], genital tract infections, increase the chances of preterm labor [22]. When it comes to assisted reproductive technologies (ARTs), it seems that frozen embryo transfers are associated with a decrease in small for gestational age and low birth weight neonates, as well as lower preterm birth rates [29-35].

The importance of identifying and assessing PBR factors has been stressed by numerous studies [19,22-24]. Early interventions by skilled obstetricians and the allocation of the necessary resources can prevent birth complications and increase the survival rate of early born babies [24]. Current risk scoring systems, though, have been disappointing, as they demonstrate low sensitivity and poor positive predictive value [19,20,36-38]. Currently, multiple logistic regression models and certain statistical methods are popular, but they are not without limitations [20,39]. They fail to test for multiple interactions among independent factors, they fall short in identifying conditions that hold true only in subgroups and they largely ignore intuition, despite its well-recognized contribution to decision making in PBR assessment. Toward addressing the challenges associated with PBR valuation, many researchers have argued the need to explore other methods such as machine learning methods, tree-based algorithms, neural networks, and fuzzy logic [20,37,39-41]. This paper suggests the development of a fuzzy multi-criteria approach to preterm risks assessment. The proposed approach addresses the multifactorial nature of the topic, integrates experts’ intuition, can identify conditions that characterize subgroups and can provide the means for the development of more effective scoring systems.

## MATERIALS AND METHODS

This section illustrates the steps of the methodology adopted for the construction of the fuzzy PBR assessment model.

### Step 1: Identification of the PBR factors

A thorough analysis of the relevant literature identified a set of risk factors. The aim was to select a comprehensive set of reasons that addresses all possible perspectives to PBR. The factors were then organized in six (6) groups, after consulting the 35 expert obstetricians who participated in this study. The six dimensions of factors reflect the multi-dimensionality of the risk assessment and they namely are: socio-economic and personal dimension, patient, behavioral and mother’s lifestyle, maternal nutrition, life habits, gynecological and obstetric history, anatomical, uterine and congenital issues, clinical medical history, reflects important aspect of pregnant women medical profile, Medical history, is related to previous pregnancies and finally, Information during Pregnancy dimension refers to issues linked to current pregnancy.

### Step 2: Data collection

The factors are subsequently organized in a hierarchy so that each dimension as identified in step 1, consists of all relevant features. The factors that are grouped under each risk dimension, are found in the literature and subsequently were approved by an expert panel. Drawing on the hierarchy, a questionnaire was designed and used to collect data from a group of 35 obstetricians, each one with more than 20 years of experience. The experts were asked to express their beliefs with respect to the relative importance of factors, by comparing them in a pairwise manner.

### Step 3: Construct the fuzzy PBR assessment model

The fuzzy analytic hierarchy process (FAHP) method was utilized to construct the proposed fuzzy evaluation model and calculate relative importance of risk factors.

### Step 4: Model Validation (I)

The proposed model was tested for its ability to produce results that are reasonable, and they reflect what is happening in the real world.

### Methods

#### FAHP

The FAHP is an extension of analytic hierarchy process (AHP) introduced by Saaty, in 1980 [42]. FAHP utilizes fuzzy logic to represent criteria with linguistic variables and their corresponding fuzzy numbers, to deal with impreciseness and vagueness in decision making.

Both the AHP and the FAHP calculate the relative importance of a set of criteria and sub-criteria, by asking experts to perform a series of pairwise criteria and sub-criteria comparisons. The consistency of the experts’ judgments is evaluated with the use of the Consistency Ratio (CR) [42]. This study calculates the CR using the modal values of fuzzy sets [43]. If CR<0.1 then responses are consistent. The extent analysis method, introduced by Jakiel and Fabianowski [44], is a popular method to solve MCDM problems with FAHP [45-47]. This research adopts the extent analysis method for it is well established and has been extensively used in many applications, even though it has been criticized for producing illogical zero weights to criteria [48,49]. To address any irrational results, this paper validates its findings by engaging experts and reassures that the shaped results are reasonable. The model derived results are compared against the judgments made by the experts and the predictive value of the proposed model is examined. Recent reviews on FAHP applications can be found in several studies [50-52]. Although fuzzy logic has been extensively used in many domains, its application in obstetrics is very limited. In preterm birth related topics, there have been studies that aim to construct instruments based on fuzzy logic that generates more reliable alarms when monitoring preterm infants [53]. Reddy et al., assess control of the infant incubator by incorporating both incubator air temperature and infant’s skin temperature to regulate the heating [54]. The potential of developing fuzzy logic systems in medical problems has been argued in many recent studies [55-60]. Indeed, it is suggested [55] that more research is needed to develop and validate fuzzy logic models in medical domains, since fuzzy logic provides the means for incorporating the subjective decision-making process in algorithms implemented by intelligent systems. The potential of fuzzy logic applications as an effective way to deal with the vagueness, uncertainty and imprecision inherited in the medical domain, is also argued [23,60].

## RESULTS

**FIGURE 1:**
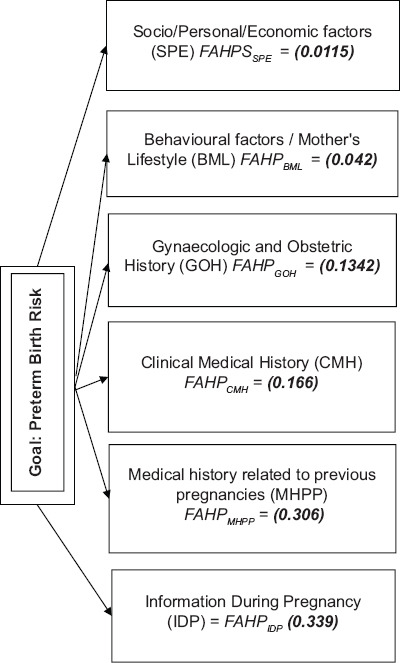
The fuzzy evaluation model of the Preterm Birth.

The FAHP is employed to calculate the relative importance of each risk factor of preterm birth. To express their knowledge and beliefs, the experts were given the linguistic scale of fuzzy sets which is shown in Figure 1. The linguistic scales and their corresponding triangular fuzzy numbers (TFNs) were adopted from Kilincci and Onal [46] and Lee et al. [61].

The expert panel participating in this study was asked to use the linguistic scales and make pairwise comparisons between the six risk dimensions, as identified in step 1, with respect to the goal, i.e., influence to PBR. Then, the linguistic scales were converted to TFNs. The consistency of the experts’ answers was evaluated by calculating the CR. The CR = 0.010536 < 0.1 indicates that the experts’ judgments are consistent.

The results show that the risk factors with the highest relative importance pertains to the Information During Pregnancy *FAHP_IIDP_* = 0.339, second highly important are factors relevant to medical history for previous pregnancies *FAHP_MHPP_* = 0.306, in the third place of importance are factors related to the clinical medical history *FAHP_CMH_* = 0.166, followed by the gynecological and obstetric history factors *FAHP_GOH_* = 0.1342, the behavioral/mother’s lifestyle factors *FAHP_BML_* = 0.042 and the Socio/Personal/Economic factors *FAHP_SPE_* = 0.0115.

FAHP analysis hierarchy and the associated importance weights are presented in Figure 1. Results in Table 1 specify that late booking and maternal age are the two most important factors by a big difference in weights. It is interesting to note that education level and marital status, although discussed in the literature, do not seem to influence the socio-economic related level of risk. A possible explanation, which needs to be investigated, is whether education and maternal age are interrelated and/or sufficiently represented by the rest of the risk factors in this dimension.

**TABLE 1:**
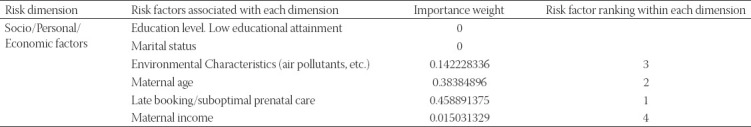
Socio/Personal/Economic factors

With respect to behavioural and mother lifestyle related factors, results show that substance use, alcohol and smoking are by far the most important top three risks that should be considered when assessing the preterm birth risk (Table 2). Table 3 indicates that all three factors included in the model influence the risk related to gynecological and obstetric risk dimension. Regarding the clinical medical history dimension, Type 1, 2 diabetes and chronic blood pressure as well as cardiovascular diseases appear to be the most significant risk causes (Table 4).

**TABLE 2:**
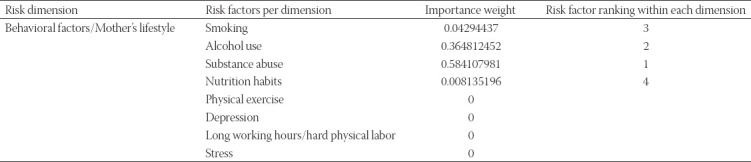
Behavioral factors/mother’s lifestyle factors

**TABLE 3:**
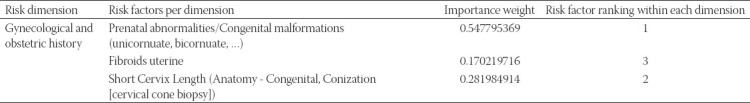
Gynecological and obstetric history factors

**TABLE 4:**
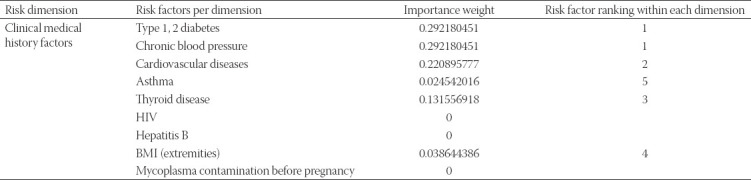
Clinical medical history factors

The previous pregnancies are reported to be significant in judging the PBR, with early gestational age and stillbirth to be the highest risk factors to be considered (Table 5). Considering both pregnant women and fetal related factors, we can see in Table 6, that early rupture of the amniotic sac and fetal fibronectin are the two top risk factors.

**TABLE 5:**
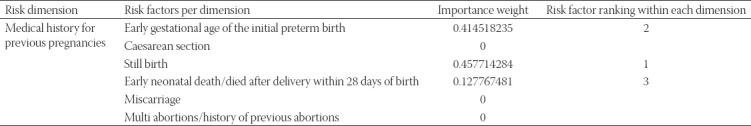
Medical history for previous pregnancies factors

**TABLE 6:**
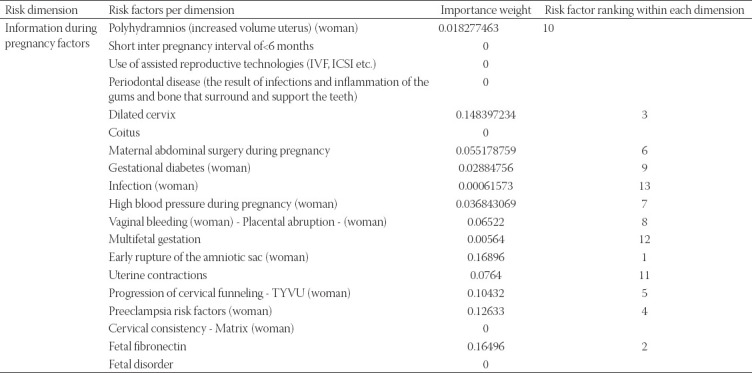
Information during pregnancy factors

### Model validation (II)

Model validation is an important step in reassuring that the results produced by the model are reasonable and they reflect what is happening in the real world.

The validation process consists of three steps:

#### First step: Expert obstetricians’ diagnosis

The group of 35 experienced obstetricians participated in this study and was asked to evaluate a set of pregnant women cases. Each case was described in terms of the risk factors considered in the proposed fuzzy model. The linguistic scale used by the experts to express their judgments, was adopted from Dawood et al. [27]. The linguistic scale and the corresponding TFNs are shown in Table 7.

**TABLE 7:**
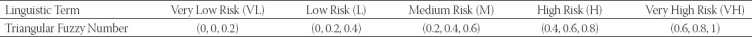
Linguistic scale used by experts to express their judgments

Expert judgments (*e_i_*), are aggregated since they are not necessarily always the same. This research uses the geometric mean to calculate the experts’ consensus, for it is assumed to represent experts’ collective judgments better than other statistical central tendency measures. This research defines TFNs to represent experts’ aggregated consensus. Thus, the aggregated TFN of the obstetricians’ responses is denoted simply as a triple e_agg_ (a, m, b), where:


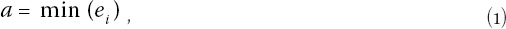


is the lowest value of all experts’ judgment, and i = 1, n represents the number of obstetricians,

(*e_i_*) represents the response of the ith obstetrician,


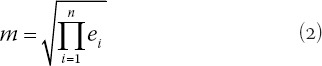


is the geometric mean of (*e_i_*), indicating the experts’ aggregated judgments, and


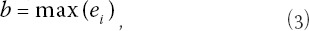


is the highest value of all experts’ judgment.

The aggregated diagnosis is subsequently fuzzified using the following (4):


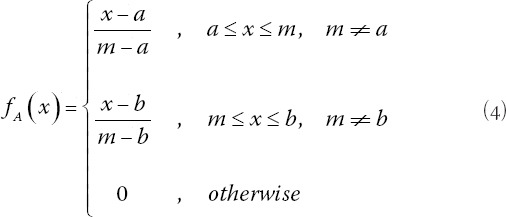


where a, m, b are real numbers. Thus, obstetricians’ responses are expressed in terms of the linguistic terms and TFNs shown in Table 8.

**TABLE 8:**
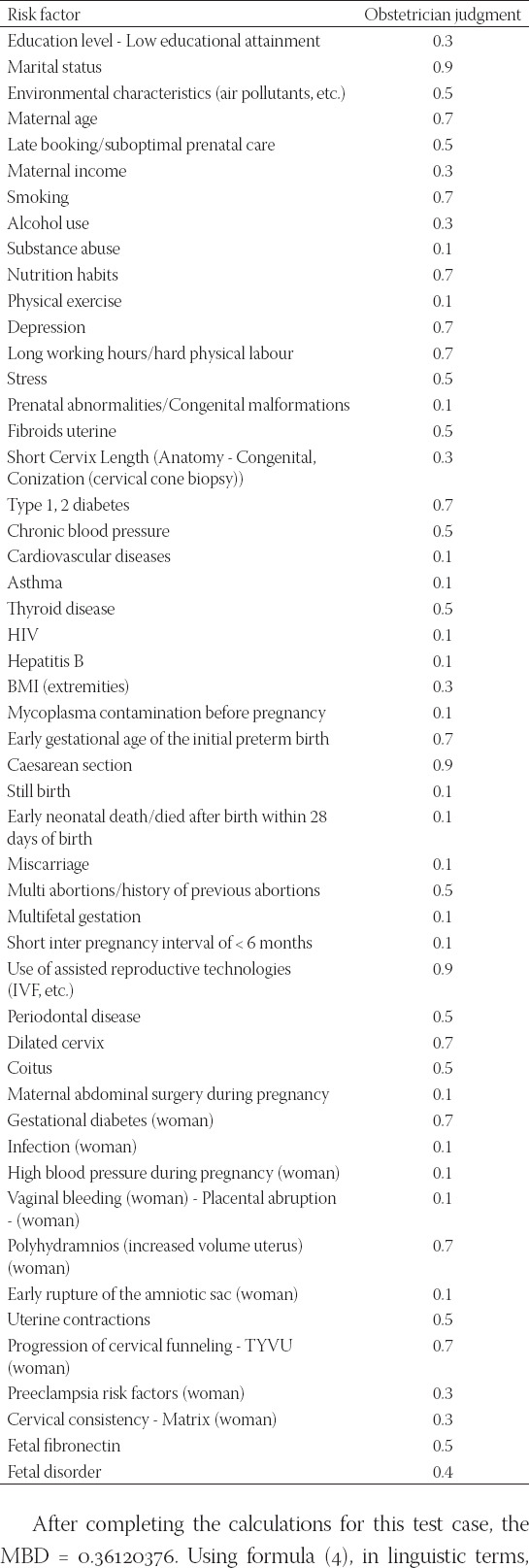
Obstetrician judgments with respect to a test case of a pregnant woman

#### Second step: Model-based diagnosis and fuzzification

The diagnosis produced by the fuzzy model (*MBD*∈[0.1]) is then calculated. The same pregnant women data set was used as input to the fuzzy model, and its results were recorded. Depending on its numerical value, the model-based diagnosis is associated with one of the linguistic terms in Table 7.

#### Third step: Investigate differences between the obstetricians and model-based diagnosis

This step compares the diagnoses proposed by the experts and the model. The results indicate the high level of predictive accuracy of the model, after examining its diagnosis accuracy with 153 carefully selected cases of pregnant women. The use of statistical method (t-test) investigates if there exists a statistical significant difference between the experts’ and the model derived diagnosis for each case. The results show that there is no statistical difference (*p* < 0.05) between the model-based diagnosis and the judgments made by the expert obstetricians. Therefore, the model is considered as valid, since it can reflect customers’ perceived satisfaction.

### Using the fuzzy model for diagnosis

Assume the following data set that represents the profile of a pregnant woman according to the requirements of the proposed fuzzy model. The obstetrician judges the individual situation (See the APPENDIX) in fuzzy linguistic terms, as shown in Table 8.

The model-based diagnosis is calculated following formula (5).


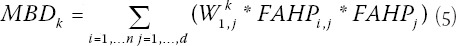


Where,

MBD_k_ is the model-based diagnosis for woman (k),

(*i*=1,…*n*,) shows the number of the risk factor,

(*j*=1,…,*d*), shows the number of risk dimensions,

is the degree associated following the obstetrician’s judgment regarding case (k), risk factor (i) of the risk dimension (j),

*FAHP_i,j_*, indicates the fuzzy model calculated weight for risk factor (i) of the risk dimension (j), and the

*FAHP_j_*, indicates the fuzzy model calculated weight the risk dimension (j).

After completing the calculations for this test case, the MBD = 0.36120376. Using formula (4), in linguistic terms, using Table 8, the membership degree for the low and medium fuzzy sets follows respectively:

*f*(*low*)=0.19 and the *f*(*medium*)=0.81. Therefore, diagnosis is medium risk.

## DISCUSSION

Subfertility appears to have an adverse effect on pregnancy outcome, independent of its treatment. A number of recent researchers have argued that women with untreated subfertility, who became pregnant, experienced adverse outcomes with higher frequency, than the general population [9,62-64]. Moreover, the complications they are faced with are as frequent as those of subfertile women who undergo ARTs [65]. All mentioned studies were observational and many potential confounders were not considered in the analyses.

More compelling support for this latter hypothesis comes from two population-based cohort studies. The first compared the pregnancy outcome of multiparous women who underwent ARTs, with the pregnancy outcome of (1) the same women in a previous or subsequent naturally conceived pregnancy, and (2) the general obstetric population [66]. Multiparous women who underwent ARTs had infants of similar gestational age and birth weight, in pregnancies before and after the procedure, but their infants were delivered earlier and had lower birth weights than the general obstetric population.

A similar population-based cohort study, that also compared siblings conceived either spontaneously or through IVF, reported that the maternal characteristic of subfertility was associated with lower birth weight, but the IVF procedure itself was not [67].

Conception by IVF, is related to an increased incidence of several obstetric and perinatal complications. Risks of preterm birth appear to be higher for fresh, as opposed to cryopreserved embryo transfers, although the magnitude of this difference is not yet clear.

The recent exploratory studies indicate that children, who were born after transfer of cryopreserved embryos, have different perinatal outcomes, than those who were born after transfer of fresh embryos [29,30,68,69]:

- Lower rates of preterm birth, low birth weight, growth restriction, and perinatal mortality
- Comparable rates of congenital malformation
- Increased rates of preeclampsia and placenta accreta spectrum

Outcome data on growth, childhood morbidity, and mental development are limited, but few differences between groups have been reported. Both slow freezing and vitrification (ultra-rapid freezing) are safe and effective methods of cryopreservation. Vitrification is greatly preferred at this time [34,70].

The reason for favorable outcomes of children born after cryopreservation, as compared with children born after fresh transfer, in most studies, is not identified. A possible explanation may be due to differences in endometrial receptivity, between women undergoing fresh versus cryopreserved embryo transfer. The lower serum E2 levels associated with frozen-thawed embryo and donor egg transfer cycles may result in better placentation. It is also possible that embryos that survive freezing and thawing are of better quality relative to fresh embryos.

## CONCLUSION

This research is to our knowledge the first study to utilize fuzzy logic and multi-criteria analysis in assessing the risk of preterm birth. The risk factors are selected after thorough literature review and consultation of a group of expert obstetricians. The model was tested against its predictive value, indicating its promising potential. The fuzzy logic approach illustrated in this study tackles the risk assessment problem by adopting a holistic perspective, integrating many different, but complementary views and allows for the obstetricians and midwives to incorporate their intuition in their judgments. Encapsulating experts’ intuition is of particular importance, especially when a full data set of the social or medical profile is not available. In addition, it can produce personalized results for individual pregnant women. In conclusion, the model can be adjusted to fit people with different ethnic backgrounds across the globe or mode of conception. It is recommended that future research should aim to further investigate the validity and the predictive value of the model. In addition, further research may combine the proposed approach with other fuzzy logic methods, use machine learning algorithms for adjusting the model hierarchy, investigate the interrelationships among reasons and calibrate risk factors weights, especially under the spectrum of assisted reproduction technologies.

## APPENDIX

**GLOSSARY**

**Cervical consistency – Matrix:** The cervix is composed of cells (e.g., smooth muscle cells, fibroblasts, glandular cells, vascular cells, and immune cells) embedded in an extracellular matrix (ECM). The ECM actively changes throughout gestation, allowing the cervix to transform from a stiff, long, and closed structure to one that is soft, short, and dilated to allow delivery.

**Cervical funneling:** A sign of cervical incompetence and represents the dilatation of the internal part of the cervical canal and reduction of the cervical length.

**Coitus:** Sexual intercourse

**Interpregnancy intervals:** Intervals between delivery and conception of the subsequent pregnancy

**Fetal fibronectin:** Is an extracellular matrix protein, normally found in fetal membranes and decidua. The presence of fetal fibronectin in the cervix or vagina, after the 20^th^ week, is abnormal.

**Multifetal gestation:** Presence of > 1 fetus in the uterus

**Polyhydramnios** **(also known as hydramnios**)**:** Refers to an excessive volume of amniotic fluid. It has been associated with an increased risk of various adverse pregnancy outcomes, including preterm birth, placental abruption, and fetal anomalies.

**Preeclampsia:** A multisystem progressive disorder characterized by the new onset of hypertension and proteinuria or the new onset of hypertension and significant end-organ dysfunction with or without proteinuria in the last half of pregnancy or postpartum.
